# Taxonomic study of the genus *Malaxa* Melichar, with descriptions of two new species from China (Hemiptera, Fulgoroidea, Delphacidae)

**DOI:** 10.3897/zookeys.861.32777

**Published:** 2019-07-08

**Authors:** Hong-Xing Li, Lin Yang, Xiang-Sheng Chen

**Affiliations:** 1 Guizhou University Guiyang China; 2 Guizhou University Guiyang China

**Keywords:** Bamboo planthopper, Fulgoromorpha, morphology, oriental region, taxonomy

## Abstract

Two new species of the delphacid genus*Malaxa* Melichar, 1914, *M.hamuliferum***sp. nov.** and *M.tricuspis***sp. nov.**, are described and illustrated from southwest China (Yunnan and Hainan), providing the genus with eleven species in total. A key is provided to distinguish the seven Chinese species in the genus.

## Introduction

The genus *Malaxa* Melichar, 1914 (Hemiptera, Auchenorrhyncha, Fulgoroidea, Delphacidae) falls within the tribe Tropidocephalini in the subfamily Delphacinae and is easily recognized from other members in this tribe by the very long antennae, and by the tegmina often with blackish brown markings (Chen et al. 2006). The Chinese species of *Malaxa* have been reviewed by Chen et al. (2006) and Hou et al. (2013). Recently, the New World species attributed to the genus was reviewed, *Malaxaoccidentalis* Muir, 1926 and *Malaxagracilis* Fennah, 1945 were transferred to *Lamaxa*Bartlett & Kennedy, 2018 (type species: *Malaxaoccidentalis* Muir, 1926), *Malaxamicrostyla* Muir, 1930 was transferred to *Xalama* Bartlett & Kennedy, 2018 (type species: *Malaxamicrostyla* Muir, 1930), and the type species *M.acutipennis* Melichar, 1914 was redescribed by Bartlett and Kennedy (2018). This genus is known to occur in the Oriental region. So far, nine species of *Malaxa* are described, including from China (five species: *M.bispinata* Muir, 1926, *M.delicata* Ding & Yang, 1986, *M.fusca* Yang & Yang, 1986, *M.hunanensis* Chen, 2006, and *M.semifusca* Yang & Yang, 1986) (Yang and Yang 1986; Ding et al. 1986; Chen et al. 2006; Ding 2006; Hou et al. 2013), Philippines (two species: *M.acutipennis* Melichar, 1914 and *M.nigra* Muir, 1919) (Melichar 1914; Muir 1919), Indonesia (two species: *M.bispinata* Muir, 1926 and *M.javanensis* Muir, 1919) (Muir 1919, 1926), Malaysia (one species: *M.obtusipennis* Muir, 1919) (Muir 1919).

Species of *Malaxa* from China with reported plant associations feed on bamboo. Specimens have been collected on leaves of bamboo in several genera, including *Bambusa*, *Indocalamus*, *Fargesia* and *Phyllostachys* (Yang and Yang 1986; Chen et al. 2006; Hou et al. 2013).

Herein, two new species: *Malaxahamuliferum* sp. nov. and *M.tricuspis* sp. nov. are described and illustrated from Hainan and Yunnan province, China. A key to species of *Malaxa* from China is provided.

## Materials and methods

The morphological terminology and measurements follow Hou et al. (2013). Body length was measured from apex of vertex to tip of tegmina. Dry male specimens were used for the description and illustration. External morphology was observed under a stereoscopic microscope and characters were measured with an ocular micrometer. Color pictures for adult habitus were obtained by the KEYENCE VHX-1000 system. The genital segments of the examined specimens were macerated in 10% KOH and drawn from preparations in glycerin jelly using a Leica MZ 12.5 stereomicroscope. Illustrations were scanned with a Canon CanoScan LiDE 200 and imported into Adobe Photoshop 6.0 for labeling and plate composition.

The type specimens of the new species are deposited in the Institute of Entomology, Guizhou University, Guiyang, China (**IEGU**).

## Taxonomy

### Malaxa

Taxon classificationAnimaliaHemipteraDelphacidae

Melichar, 1914

Malaxa Melichar, 1914: 275; Muir 1926: 7; Metcalf 1943: 103; Fennah 1945: 429; Yang and Yang 1986: 56; Ding et al. 1999: 443; Chen et al. 2006: 160; Ding 2006: 150; Bartlett 2009: 387; Hou et al. 2013: 864; Bartlett and Kennedy 2018: 514.

#### Type species.

*Malaxaacutipennis* Melichar, 1914.

#### Diagnosis.

Description from Hou et al. (2013: 286–287) “Body slender and elongate, length (from apex of vertex to tip of tegmina): male 3.7–4.8 mm, female 4.3–5.1 mm, often with blackish brown markings. Head with eyes narrower than pronotum. Vertex longer or slightly shorter in middle than broad at base (0.95–1.24: 1), apex projected in front of eyes. Submedian carinae uniting before apex, greatest length of basal compartment shorter than wide at base of vertex (0.48–0.81: 1). Frons relatively long, longer in middle line than wide at widest part (~ 2.73–3.00: 1), widest at middle or apex. Rostrum reaching mesothoracic trochanters. Antennae cylindrical, very long, surpassing apex of clypeus, basal segment longer in middle than wide at apex (3.67–5.22: 1), shorter than frons in middle line (0.49–0.74: 1), shorter than the second segment (0.40–0.56: 1). Pronotum shorter than vertex in middle line (0.58–0.96: 1), lateral carinae attaining hind margin. Mesonotum longer in middle line than vertex and pronotum together (1.33–2.05: 1). Tegmina elongate, longer in middle line than wide at widest part (1.76–3.16: 1), much longer than abdomen, hyaline, cross vein deposited medially, apical margin acutely rounded. Spinal formula of hind tibia 5-6-4. Post-tibial spur large and thick, concave on inner surface, without teeth along the hind margin, with an apical tooth. Anal segment of male short, ring-like, left lateroapical angle produced into process. Pygofer with two broad lamellate medioventral processes, between of them with a V-like emargination. Genital styles broad in basal half, forked or with process at apex. Aedeagus with or without phallobase, phallus tubular, curved C-like and directed segmental venter.”

##### Key to species (males) of *Malaxa* from China *(revised from Hou et al. 2013)*

**Table:** 

1	Postclypeus yellow; tegmina with apical veins Cu_1_ and M_3_ diverging apically, posterior half of apical tegmina dark brown (see Chen et al. 2006: figs 2, 3)	*** M. semifusca ***
–	Postclypeus with basal half blackish brown; tegmina with apical veins Cu_1_ and M_3_ fused, first and second apical cells hyaline	**2**
2	Anal segment of male without process; aedeagus with phallobase	**3**
–	Anal segment of male with a long process; aedeagus without phallobase	**4**
3	Genae with basal half dark brown, apical half yellowish white; tegmina mostly hyaline; pygofer with 3 medioventral processes very distinct; genital styles with inner margin without process (see Hou et al. 2013: figs 2, 3, 8–11)	*** M. bispinata ***
–	Genae all dark brown; tegmina with basal half most yellow, apical half most dark brown; pygofer with medioventral processes not distinct; genital styles with inner margin with large process medially, hook-like (Figs 7–9, 11, 13)	***M* . *hamuliferum* sp. nov.**
4	Genae dark brown; in posterior view, process of anal segment of male situated in middle of ventral margin (Chen et al. 2006: figs 21, 23, 25)	*** M. hunanensis ***
–	Genae mostly dark brown but apical with small part yellow; in posterior view, process of anal segment of male situated on left side of ventral margin	**5**
5	Genital styles with apex not forked; aedeagus with three processes (Figs 22–24)	***M.tricuspis* sp. nov.**
–	Genital styles with apex forked; aedeagus with two processes	**6**
6	Area between lateral carinae of pronotum dark brown; two branches of outer apical angle of genital styles subequal; aedeagus with a small spine situated near basal third, directed caudally (see Chen et al. 2006: figs 30, 37, 38)	*** M. delicata ***
–	Area between lateral carinae of pronotum mostly yellow; two branches of outer apical angle of genital styles unequal; aedeagus with a small tooth situated near middle, directed right (see Chen et al. 2006: figs 11, 18, 19)	*** M. fusca ***

### Malaxa hamuliferum sp. nov.

Taxon classificationAnimaliaHemipteraDelphacidae

http://zoobank.org/590A3548-6436-40FB-91F0-3A1CB1CD0237

Figs 1 , 2 , 5–14

#### Type material.

Holotype: ♂, **China**: Yunnan, Yingjiang County (24°44'N, 97°33'E), on bamboo, 17 August 2018, Hong-Xing Li; paratypes, 5♂♂, 10♀♀, same data as holotype, Hong-Xing Li and Qiang Luo.

#### Etymology.

The specific name is derived from the Latin word “*hamulus*” and the postfix “-*ferus*”, referring to the middle of genital styles with large process, hook-like.

#### Measurements.

Body length including tegmina: male 3.9–4.1 mm (*N* = 10); female 4.8–5.0 mm (*N* = 5); tegmen length: male 3.4–3.6 mm (*N* = 10); female 4.0–4.4 mm (*N* = 5).

#### Diagnosis.

The salient features of the new species include the following: aedeagus with phallobase broad basally, apical third narrowing abruptly, and genital styles with large process at middle, hook-like.

#### Description.

**Figures 1–4.**
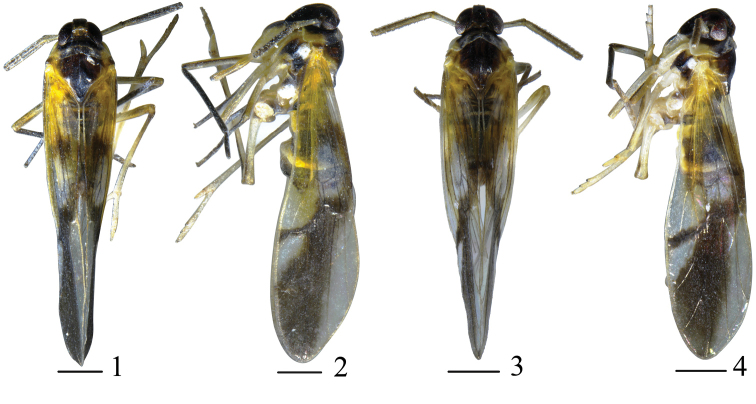
*Malaxahamuliferum* sp. nov. **1** male habitus, dorsal view **2** same, lateral view **3–4***&nbsp;Malaxa&nbsp;tricuspis* sp. nov. **3** male habitus, dorsal view **4** same, lateral view. Scale bars: 0.5 mm.

**Figures 5–14.**
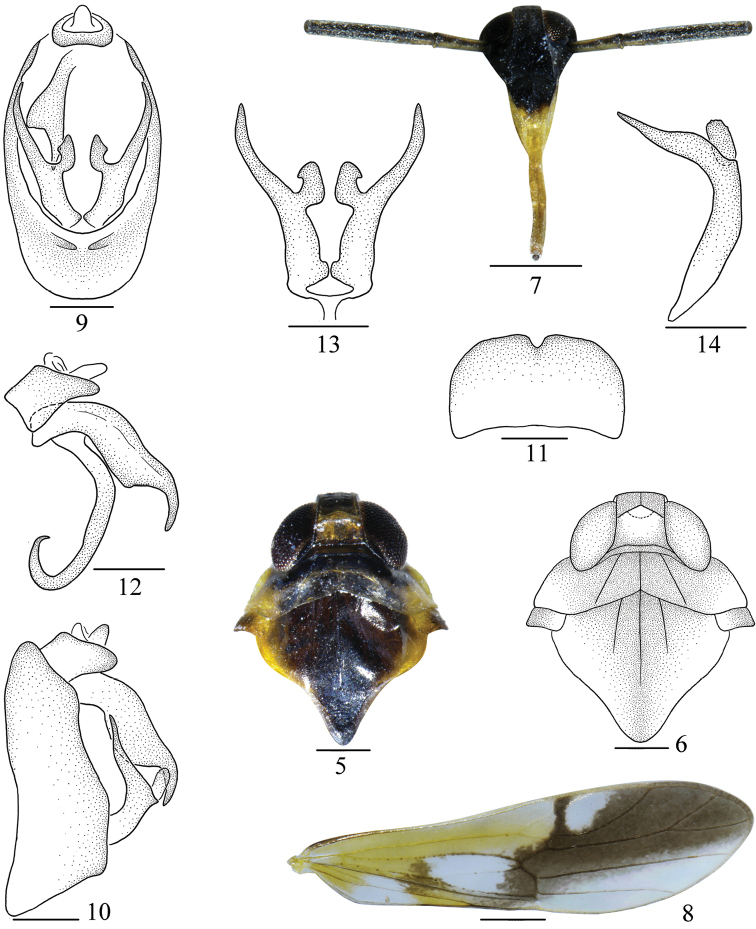
*Malaxahamuliferum* sp. nov. **5** head and thorax, dorsal view **6** same **7** face **8** tegmen **9** male genitalia, posterior view **10** same, lateral view **11** pygofer, ventral view **12** anal segment and aedeagus, lateral view **13** genital style, posterior view **14** same, left lateral view. Scale bars: 0.5 mm (**5–8**); 0.2 mm (**9–14**).

*Coloration*. General color pale yellowish brown, with dark brown to black markings, shiny (Figs 1, 2). Vertex with basal half yellowish brown, apical half pale black. Pronotum and mesonotum brown to black except each lateral side yellow (Figs 5, 6). Frons and genae black. Clypeus with basal half black, rest yellow. Rostrum yellow except apex pale brown (Fig. 7). Eyes and ocelli reddish brown. First segment of antennae with dorsal side pale yellow, with ventral side brown, second segment dark brown. Tegmina with basal half yellow except areas around apex of Cu_1_, after bifurcation of IA and IIA hyaline, at apical half, along Sc_1_, sc-r and area between R_1_ and M_2_ dark brown (Fig. 8). Wings hyaline, veins brown. Abdomen with dorsal side black, with ventral side yellow white. Genitalia dark brown.

*Head and thorax*. Vertex (Figs 5, 6) longer submedially than wide at base ~ 0.98: 1, at base longer than at apex ~ 1.4: 1, submedian carinae uniting slightly beyond middle, apex produced in front of eyes, apical margin straight, greatest length of basal compartment shorter than wide at base of vertex ~ 0.53: 1. Frons (Fig. 7) longer in middle line than wide at widest part ~ 1.47: 1, widest at apex. Postclypeus wide at base as wide as frons at apex. Antennae very long, cylindrical, surpassing apex of clypeus, first segment longer than wide ~ 4.06: 1, shorter than frons in middle line ~ 0.79: 1, shorter than the second segment ~ 0.43: 1 (Fig. 7). Pronotum with lateral carinae not attaining hind margin, shorter than vertex ~ 0.69: 1. Mesonotum with lateral carinae not attaining hind margin, longer in middle line than vertex and pronotum together ~ 1.76: 1 (Figs 5, 6). Tegmina narrow, longer than widest part ~ 4.12: 1 (Fig. 8).

*Male genitalia*. Anal segment of male small, ring-like (Fig. 9). Pygofer in profile tapering to dorsad (Fig. 10), in posterior view with opening longer than wide (Fig. 9), in ventral view medioventral margin V-like (Fig. 11). Aedeagus with phallus slender, tubular, acute at apex, apical third curved C-like, phallobase in profile broad basally, apical third narrowing abruptly (Fig. 12). Genital styles long and slender, tapering to apex, inner margin with large process at middle, hook-like (Fig. 13).

#### Host plant.

Bamboo.

#### Distribution.

Southwest China (Yunnan).

#### Remarks.

This species is similar to *Malaxasemifusca* Yang & Yang, 1986 but differs from it by: (1) frons and genae black, clypeus with basal half black (frons with apical third, genae with ventral half and clypeus yellow in *M.semifusca*); (2) anal segment of male without process (anal segment with left lateroapical process small and obtuse in *M.semifusca*); (3) aedeagus with phallobase without tooth at apex (aedeagus with phallobase incomplete, apex membraneous, with several teeth along margin and around apex in *M.semifusca*).

### Malaxa tricuspis sp. nov.

Taxon classificationAnimaliaHemipteraDelphacidae

http://zoobank.org/D71D3BB4-F1DC-4030-926A-1270252C7532

Figs 3 , 4 , 15–24

#### Type material.

Holotype: ♂, **China**: Hainan, Wanning County (18°55'N, 110°20'E), on bamboo, 6 May 2017, Hong-Xing Li; paratypes, 6♂♂, 8♀♀, same data as holotype.

#### Etymology.

The specific name is derived from the Latin word “*tricuspis*”, referring to aedeagus with three small processes.

#### Measurements.

Body length including tegmina: male 3.5–3.7 mm (*N* = 7); female 4.1–4.3 mm (*N* = 8); tegmen length: male 3.0–3.2 mm (*N* = 7); female 3.5–3.8 mm (*N* = 8).

#### Diagnosis.

The salient features of the new species include the following: left lateroapical process of anal segment stout and twisted, tapering apically; aedeagus with three small processes.

#### Description.

**Figures 15–24.**
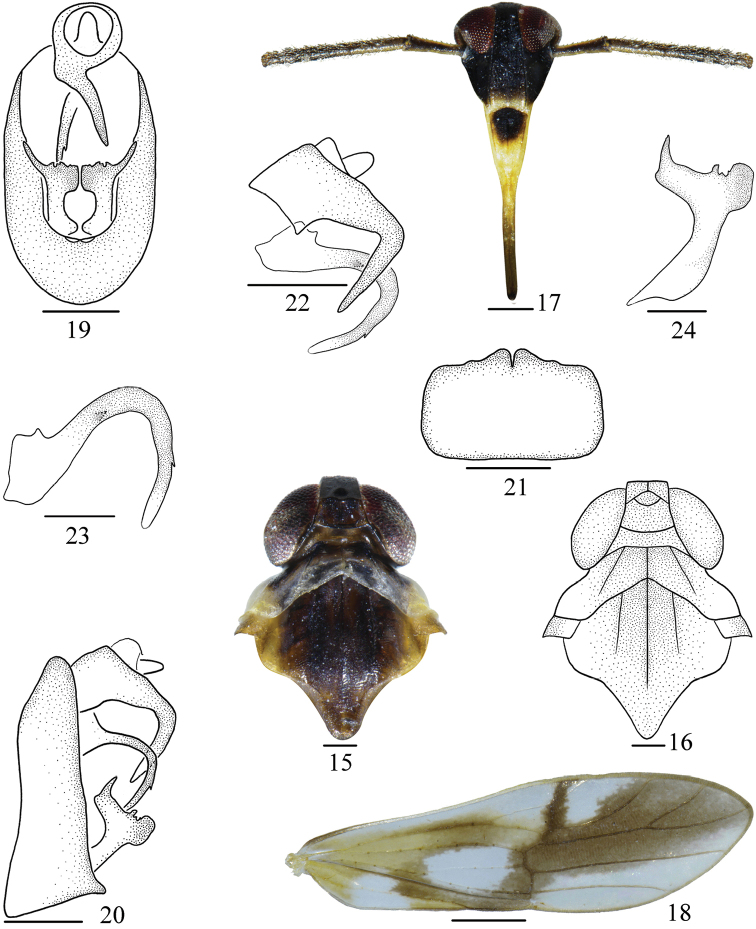
*Malaxatricuspis* sp. nov. **15** head and thorax, dorsal view **16** same **17** face **18** tegmen **19** male genitalia, posterior view **20** same, lateral view **21** pygofer, ventral view **22** anal segment and aedeagus, lateral view **23** aedeagus **24** genital style, left lateral view. Scale bars: 0.5 mm (**15–18**); 0.2&nbsp;mm&nbsp;(**19–22**); 0.1 mm (**23, 24**).

*Coloration*. General color pale yellowish brown, with dark brown to black markings, shiny (Figs 3, 4). Vertex, pronotum and mesonotum pale black except each lateral side yellow (Figs 15, 16). Frons and genae black except small area at apex yellow. Clypeus with basal half black, rest yellow (Fig. 17). Eyes and ocelli reddish brown. Antennae with dorsal side pale yellow, with ventral side brown. Tegmina with basal half pale yellow except areas around Sc+R, apex of Cu_1_, after bifurcation of IA and IIA hyaline, at apical half, along Sc_1_, sc-r, and area between R_1_ and M_2_ dark brown (Fig. 18). Wings hyaline, veins brown. Abdomen with dorsal side black, with ventral side yellow. Genitalia brown.

*Head and thorax*. Vertex (Figs 15, 16) longer submedially than wide at base ~ 0.91: 1, at base longer than at apex ~ 1.65: 1, forming a circular cell, submedian carinae uniting slightly beyond middle, apex produced in front of eyes, apical margin straight, greatest length of basal compartment shorter than wide at base of vertex ~ 0.55: 1. Frons (Fig. 17) longer in middle line than wide at widest part ~ 2.83: 1, widest at near apex. Postclypeus wide at base as wide as frons at apex. Antennae very long, cylindrical, surpassing apex of clypeus, first segment longer than wide ~ 4.44: 1, shorter than frons in middle line ~ 0.57: 1, shorter than the second segment ~ 0.41: 1 (Fig. 17). Pronotum with lateral carinae not attaining hind margin, shorter than vertex ~ 0.55: 1. Mesonotum with lateral carinae not attaining hind margin, longer in middle line than vertex and pronotum together ~ 2.06: 1 (Figs 15, 16). Tegmina narrow, longer than widest part ~ 3.28: 1 (Fig. 18).

*Male genitalia*. Anal segment of male small, ring like, left lateroapical process stout and twisted, tapering to apex (Fig. 19). Pygofer in profile tapering to dorsad, ventral angles strongly produced (Fig. 20), in posterior view with opening longer than wide (Fig. 19), in ventral view medioventral processes wide, concave medially (Fig. 21). Aedeagus simple, tubular, broad basally then tapering to apex, with stout process at base, a spine at basal third and with small tooth at apical third (Figs 22, 23). Genital styles long, broad basally, apical half narrowing abruptly, inner margin with several teeth at middle (Figs 19, 24).

#### Host plant.

Bamboo.

#### Distribution.

Southwest China (Hainan).

#### Remarks.

This species is similar to *Malaxafusca* Yang & Yang, 1986 but differs from it by: (1) anal segment of male with left lateroapical process twisted but not S-like, not swelled subapically (anal segment with left lateroapical process twisted, S-like, swelled subapically in *M.fusca*); (2) aedeagus with stout process at base, a spine at basal third and with small tooth at apical third (aedeagus with small process at base and with a spine near middle in *M.fusca*); (3) genital styles with apical half narrowing abruptly, not forked at apex (genital styles with outer angle forked at apex, inner branch longer than outer one in *M.fusca*).

This species is also similar to *M.delicata* Ding & Yang, 1986 but differs from it by: (1) anal segment of male with left lateroapical process twisted near base (anal segment with left lateroapical process twisted near apex in *M.delicata*) (2) aedeagus with stout process at base, a spine at basal third and with small tooth at apical third (aedeagus with process at base and with small spine at basal third in *M.delicata*); (3) genital styles with outer angle not forked at apex (genital styles with outer angle forked at apex, two branches subequally long in *M.delicata*).

## Discussion

Melichar (1914) established the genus *Malaxa* with the type species *M.acutipennis* Melichar, 1914 from Philippines. This genus is only known to occur in the Oriental region, with highest species density occurring in China. Bartlett and Kennedy (2018: 515–516) noted “Several differences can be observed between *Malaxaacutipennis* and the Chinese species in that genus. The most salient of these are that *M.acutipennis* has an apically pointed forewing (rounded in all other *Malaxa*) with the leading margin arced (giving the wing a spatulate appearance; parallel-sided in all other *Malaxa*); the more elongate pronotum with the carinae clearly reaching the hind margin (most other *Malaxa* with a relatively shorter pronotum with lateral carinae not reaching); and the genitalia with a simple ventral margin of the pygofer opening (vs. having projections on the opening of the pygofer); and the simple anal tube (most Chinese *Malaxa* bear a single, large, asymmetrical projection on the anal tube).”

The *Malaxa* species distributed in China with common type characters: body slender and elongate, often with blackish brown markings; antennae cylindrical, very long, surpassing apex of clypeus, basal segment shorter than the second segment (0.40–0.56: 1); tegmina apically rounded, leading margin straight; anal tube of male either simple or with 1–2 processes; opening of pygofer usually bearing two broad lamellate medioventral processes, between them a V-like emargination; genital styles broad in basal half, forked or with process at apex; aedeagus with or without phallobase, phallus tubular, curved C-like and directed segmental venter. Based mainly on the characters of the morphological and male genitalia, we also found obvious differences between the Chinese *Malaxa* and the type species *M.acutipennis*, which agrees Bartlett and Kennedy’s (2018) description. Therefore, the genus level composition of&nbsp;*Malaxa*&nbsp;may require reconsideration, with the general concern that the Chinese species may not be congeneric with the type species from the Philippines. However, *M.obtusipennis* Muir, 1919 (from Malaysia: Sabah) was described from three females. The features of the male terminalia of *M.obtusipennis* are not available for consideration, which limits our ability to place this species. Therefore, in this paper, we provisionally place the two new species in the genus *Malaxa*, but the genus level composition of&nbsp;*Malaxa* is required to reconsideration and more taxon samples or molecular data are still required to confirm the relationships within *Malaxa* in the future.

## Supplementary Material

XML Treatment for Malaxa

XML Treatment for Malaxa hamuliferum

XML Treatment for Malaxa tricuspis

## References

[B1] Bartlett CR (2009) A new genus of New World Tropidocephalini (Hemiptera: Delphacidae: Delphacinae), with the description of two new species. Entomological News 120(4): 387–396. 10.3157/021.120.0407

[B2] Bartlett CR Kennedy AC (2018) A review of New World *Malaxa* (Hemiptera: Fulgoroidea: Delphacidae). Zootaxa 4441(3): 511–528. 10.11646/zootaxa.4441.3.5 30313998

[B3] Chen XS Li XF Liang AP Yang L (2006) A review of the bamboo delphacid genus *Malaxa* (Hemiptera: Fulgoroidea: Delphacidae) from China. Annales Zoologici 56(1): 159–166.

[B4] Ding JH Yang LF Hu CL (1986) Descriptions of new genera and species of Delphacidae attacking bamboo from Yunnan Province, China. Acta Entomologische Sinica 29(4): 415–425.

[B5] Ding JH (2006) Fauna Sinica. Insecta Vol. 45. Homoptera, Delphacidae. Science Press, Beijing, 150–154.

[B6] Ding JH Zhuo WX Huang BK (1999) Delphacidae of Fujian (Homoptera: Fulgoroidea). In: Huang BK (Ed.) Fauna of Insects in Fujian Province of China.Vol. 2. Fujian Science and Technology Press, Fuzhou, 432–464.

[B7] Fennah RG (1945) New lanternflies (Fulgoroidea) from South America. Proceedings of the United States National Museum 96(3189): 95–106. 10.5479/si.00963801.96-3189.95

[B8] Hou XH Yang L Chen XS (2013) A checklist of the genus *Malaxa* (Hemiptera: Fulgoromorpha: Delphacidae) with descriptions and illustrations of *Malaxabispinata* newly recorded in China and the fifth instar of *Malaxadelicata*. Florida Entomologist 96(3): 864–870. 10.1653/024.096.0321

[B9] Melichar L (1914) Neue Fulgoriden von den Philippinen: I. Theil. Philippine Journal of Science 9(3): 269–283.

[B10] Metcalf ZP (1943) General Catalogue of the Hemiptera. Fascicle IV, Fulgoroidea, Part 3, Araeopidae (Delphacidae). Smith College, Northhampton, Massachusetts, 551 pp.

[B11] Muir F (1919) Some Malayan Delphacidae (Homoptera). Philippine Journal of Science 15(6): 521–531.

[B12] Muir F (1926) Contributions to our knowledge of South American Fulgoroidea (Homoptera). Part I. The Family Delphacidae. Experiment Station of the Hawaiian Sugar Planters’ Association, Entomological Series, Bulletin 18: 1–51.

[B13] Muir F (1930) On some South American Delphacidae (Homoptera, Fulgoroidea). Entomologisk Tidskrift 51: 207–215.

[B14] Yang JT Yang CT (1986) Delphacidae of Taiwan (1) Asiracinae and the tribe Tropidocephalini (Homoptera: Fulgoroidea). Taiwan Museum Special Publication Series 6: 1–79.

